# AMnO_3_ (A = Sr, La, Ca, Y) Perovskite Oxides as Oxygen Reduction Electrocatalysts

**DOI:** 10.1007/s11244-018-0886-5

**Published:** 2018-01-16

**Authors:** V. Celorrio, L. Calvillo, G. Granozzi, A. E. Russell, D. J. Fermin

**Affiliations:** 1 School of Chemistry, University of Bristol, Cantocks Close, Bristol, BS8 1TS UK; 2 Dipartimento di Scienze Chimiche, Università di Padova, Via Marzolo 1, 35131 Padua, Italy; 3 School of Chemistry, University of Southampton, Highfield, Southampton, UK

**Keywords:** Perovskite, Manganite oxide, A-site, Oxygen reduction reaction, Catalytic activity

## Abstract

A series of perovskite-type manganites AMnO_3_ (A = Sr, La, Ca and Y) particles were investigated as electrocatalysts for the oxygen reduction reaction. AMnO_3_ materials were synthesized by means of an ionic-liquid method, yielding phase pure particles at different temperatures. Depending on the calcination temperature, particles with mean diameter between 20 and 150 nm were obtained. Bulk versus surface composition and structure are probed by X-ray photoelectron spectroscopy and extended X-ray absorption fine structure. Electrochemical studies were performed on composite carbon-oxide electrodes in alkaline environment. The electrocatalytic activity is discussed in terms of the effective Mn oxidation state, A:Mn particle surface ratio and the Mn–O distances.

**Electronic supplementary material:**

The online version of this article (10.1007/s11244-018-0886-5) contains supplementary material, which is available to authorized users.

## Introduction

Electrocatalysts that can support the oxygen reduction reaction (ORR) at high current densities are key components of electrochemical energy conversion systems such as metal/air secondary batteries and fuel cells [1–4]. It is generally considered that ORR can proceed via a four electron transfer to produce water (pathway 1), or via a two electron transfer to produce H_2_O_2_ (pathway 2) [5, 6].

1 $${{\text{O}}_2}+2{{\text{H}}_2}{\text{O}}+4{{\text{e}}^ - } \to 4{\text{O}}{{\text{H}}^ - }$$

2 $${{\text{O}}_2}+{{\text{H}}_2}{\text{O}}+2{{\text{e}}^ - } \to {\text{H}}{{\text{O}}_2}^{ - }+{\text{O}}{{\text{H}}^ - }$$

The generation of OH^−^ from H_2_O_2_ can be achieved by either a further reduction reaction (pathway 3),

3 $${\text{H}}{{\text{O}}_2}^{ - }+{{\text{H}}_2}{\text{O}}+2{{\text{e}}^ - } \to 3{\text{O}}{{\text{H}}^ - }$$

or the decomposition step

4 $$2{\text{H}}{{\text{O}}_2}^{ - } \to 2{\text{O}}{{\text{H}}^ - }+{{\text{O}}_2}$$

Platinum-based catalysts remain the benchmark materials for the ORR, in acidic and alkaline environments, but significant efforts have more recently been focused on earth-abundant and lower-cost materials; such as perovskite oxides of the general structure ABO_3_ [7–9]. Significant efforts have been devoted to establishing electronic descriptors for rationalising and predicting the activity of this class of materials [10–13]. Our recent studies have established links between changes in the redox state of the B-site and their activity towards ORR which, in the case of Mn based oxides, can occur at potentials close to the reversible oxygen potential [14–16]. We proposed that this is the key rational behind the high activity of LaMnO_3+δ_ observed by many groups [17, 18].

Several studies have also focused on the effect of the A-site in AMnO_3_ structures, including Pr_1−x_A_x_ (A = Ca, Sr, Ba) [19], La_1−x_Ca_x_ [14], La_1−x_Sr_x_ [20], La_1−x_Te_x_ [16] and A = Lanthanide element (La, Pr, Nd, Sm, Gb, Y, Dy and Yb) [21]. However, rationalising trends for different synthetic approaches and experimental conditions is rather complex due to the contribution of other phenomena such as A-site surface segregation [22] and oxygen vacancies [23, 24]. In this work, we perform a systematic study of the ORR activity of AMnO_3_ particles as a function of the nature of the A-site (Ca, Sr, La and Y). A common synthetic strategy is implemented to generate phase pure particles which are characterised by X-ray diffraction (XRD), X-ray photoemission spectroscopy (XPS) and X-ray absorption spectroscopy (XAS). Electrochemical studies of the oxide particles mixed with mesoporous carbon and deposited at a rotating ring-disc electrode suggest that overall activity and selectivity towards the four-electron ORR reaction show complex dependence on structural parameters such as the ionic radius of the A cation and the average Mn–O distance.

## Materials and Methods

### Synthesis and Characterization of the Materials

The perovskite oxide particles were synthesized employing a route described in previous works [14–16, 25, 26]. The synthesis involved the dispersion of Mn(NO_3_)_3_ (0.5 mL, 0.1 M in H_2_O) and 0.5 mL of the corresponding A-site metal nitrate (0.5 mL, 0.1 M in H_2_O) in 1-ethyl-3-methylimidazolium acetate (1 mL) under stirring conditions. After heating at 95 °C for 4 h to ensure all water was removed, cellulose (100 mg) is added and the mixture was stirred for 20 min. The gel-like mixture was calcined for 4 h, except LaMnO_3_ and CaMnO_3_ which were calcined for 2 h. The calcination temperatures for each material were as follows: 850 °C (CaMnO_3_), 950 °C (LaMnO_3_, BaMnO_3_ and SrMnO_3_) and 1100 °C for YMnO_3_. Calcination conditions were set to ensure single-phase formation.

Powder X-ray diffraction (XRD) was carried out on a Bruker D8 Advance using Cu Kα radiation. Experiments were run between 10° and 90°, using a step size of 0.02°. Scanning electron microscopy (SEM) was carried out on a JEOL SEM 5600LV scanning electron microscope. Transmission electron microscopy (TEM) was carried out on a JEOL JEM-1400Plus instrument.

X-ray photoemission spectroscopy (XPS) measurements were performed in a custom-designed UHV system equipped with an EA 125 Omicron electron analyser, working at a base pressure of 10^−10^ mbar. Core level photoemission spectra were collected in normal emission at room temperature with a non-monochromatized Al K_α_ X-ray source (1486.7 eV), using 0.1 eV steps, 0.5 s collection time and 20 eV pass energy. The binding energies (BE) were referenced to the C 1s peak at 284.6 eV.

XAFS spectra were recorded in transmission mode at the Mn K-edge (6539 eV), on beamline B18 at Diamond Light Source (UK) operating with a ring energy of 3 GeV and at a current of 300 mA. The monochromator comprises Si(311) crystals operating in Quick EXAFS mode. Calibration of the monochromator was carried out using a Mn foil. The samples were prepared as pellets by mixing the ground sample with cellulose to form a homogeneous mixture. A total of three spectra were averaged for each sample. The spectra were aligned using the Mn foil response. The data was analysed using the Athena and Arthemis software [27], which implement the FEFF6 and IFEFFIT codes [28]. Fits were carried out using a *k* range of 3.5–12 Å^−1^ and a *R* range of 1.0–4 Å with multiple *k* weightings of 1, 2 and 3. To perform the fittings, the coordination numbers were fixed to the crystallographic values; interatomic distances and Debye–Waller factors were fitted.

### Electrochemical Measurements

A three-electrode cell was used to conduct the electrochemistry experiments, using a rotating ring-disk electrode (RRDE) fitted to an ALS rotation controller and connected to a CompactStat bipotentiostat (Ivium). The RRDE electrode consisted of a 4 mm glassy carbon disk surrounded by a Pt ring; a graphite rod and a Hg/HgO (in 0.1 M NaOH) were used as counter and reference electrodes, respectively. Measurements were recorded in 0.1 M KOH solution saturated with either purified Ar or O_2_. A two-step drop-casting process was used to prepare the working electrodes. First, an aqueous Vulcan/Nafion suspension was dropped onto the surface of the electrode, followed by an aqueous suspension of the metal oxide nanoparticles. The sample loading on the electrode surface was 250 µg_Oxide_ cm^−2^, 50 µg_Vulcan_ cm^−2^, and 50 µg_Nafion_ cm^−2^.

## Results and Discussion

**Fig. 1:**
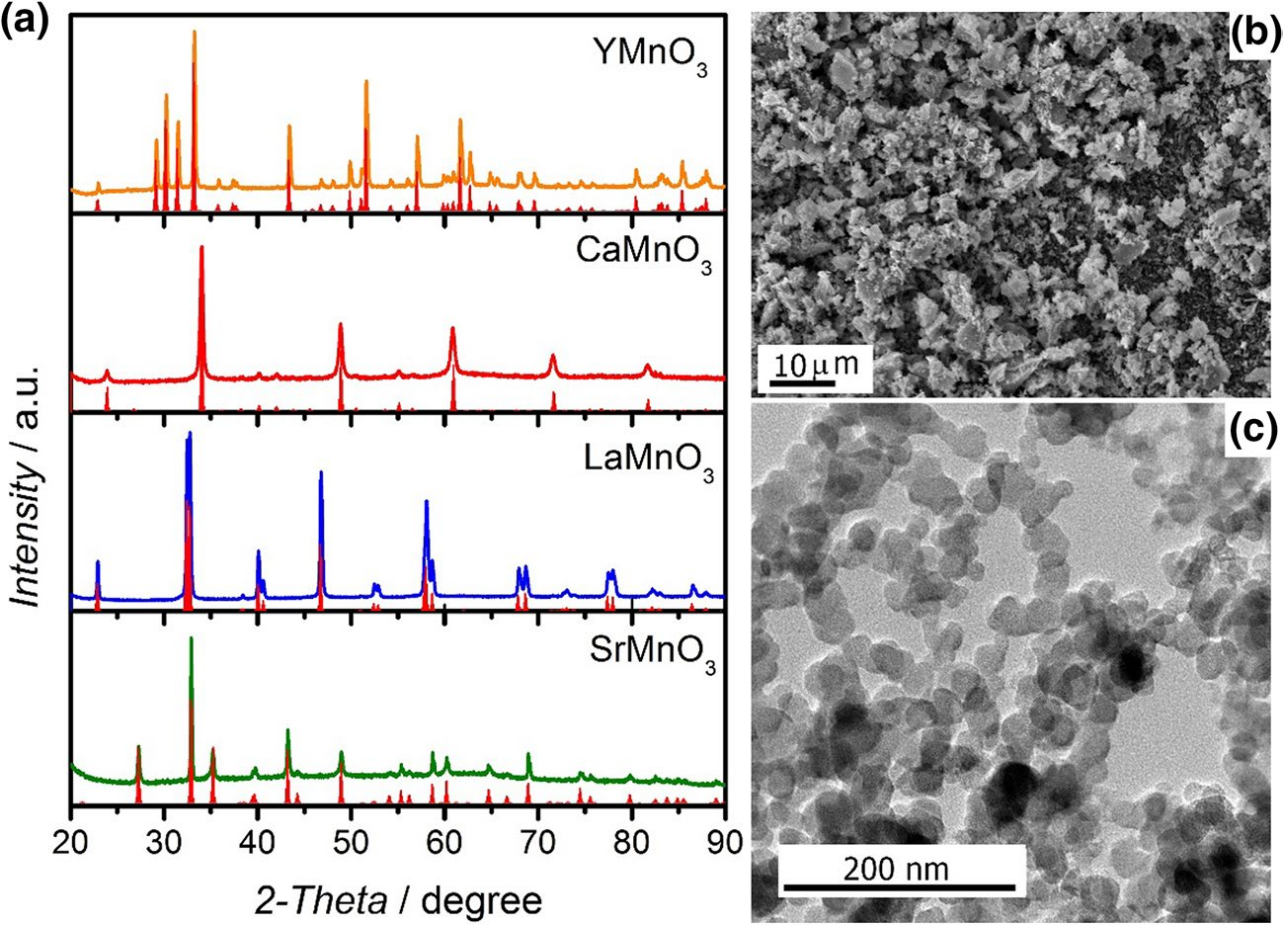
**a** XRD patterns of SrMnO_3_, LaMnO_3_, CaMnO_3_, and YMnO_3_. Red vertical bars correspond to standard patterns, JCPDS-ICDD File Nos. 01-072-0197, 01-085-0372, 01-076-1132, and ICSD Code 191978, respectively. **b** SEM and **c** TEM images of as-prepared CaMnO_3_

Figure 1a shows the XRD patterns of the as-grown AMnO_3_ (A = Y, Ca, La and Sr) oxide nanoparticles, revealing a high degree of phase purity based on their close match with the reported standards. YMnO_3_ was indexed to the hexagonal *P*6_3_*cm* structure characterized by tilted layers of corner-linked MnO_5_ trigonal bipyramids, separated by layers of Y [29]; whereas SrMnO_3_ was indexed to the *P*6_3_/*mmc* hexagonal space group. LaMnO_3_ was indexed to the rhombohedral (*R-3c*) phase, while CaMnO_3_ exhibited the orthorhombic (*Pnma*) phase.

Figure 1b displays a representative SEM image of CaMnO_3_ illustrating the microstructure of the material, while the TEM image in Fig. 1c shows the nanoscale dimensions of the particles (TEM images of the other oxides can be found in Fig. S1 of the Supporting Information). Particle size distributions (Fig. S2) revealed an increase in the mean particle size as the synthesis temperature increases, from 25.9 ± 4.3 nm for CaMnO_3_ to 166.1 ± 28.5 nm for YMnO_3_. Specific surface areas calculated from the mean diameters as measured by TEM and the theoretical densities are summarised in Table S1.

**Fig. 2:**
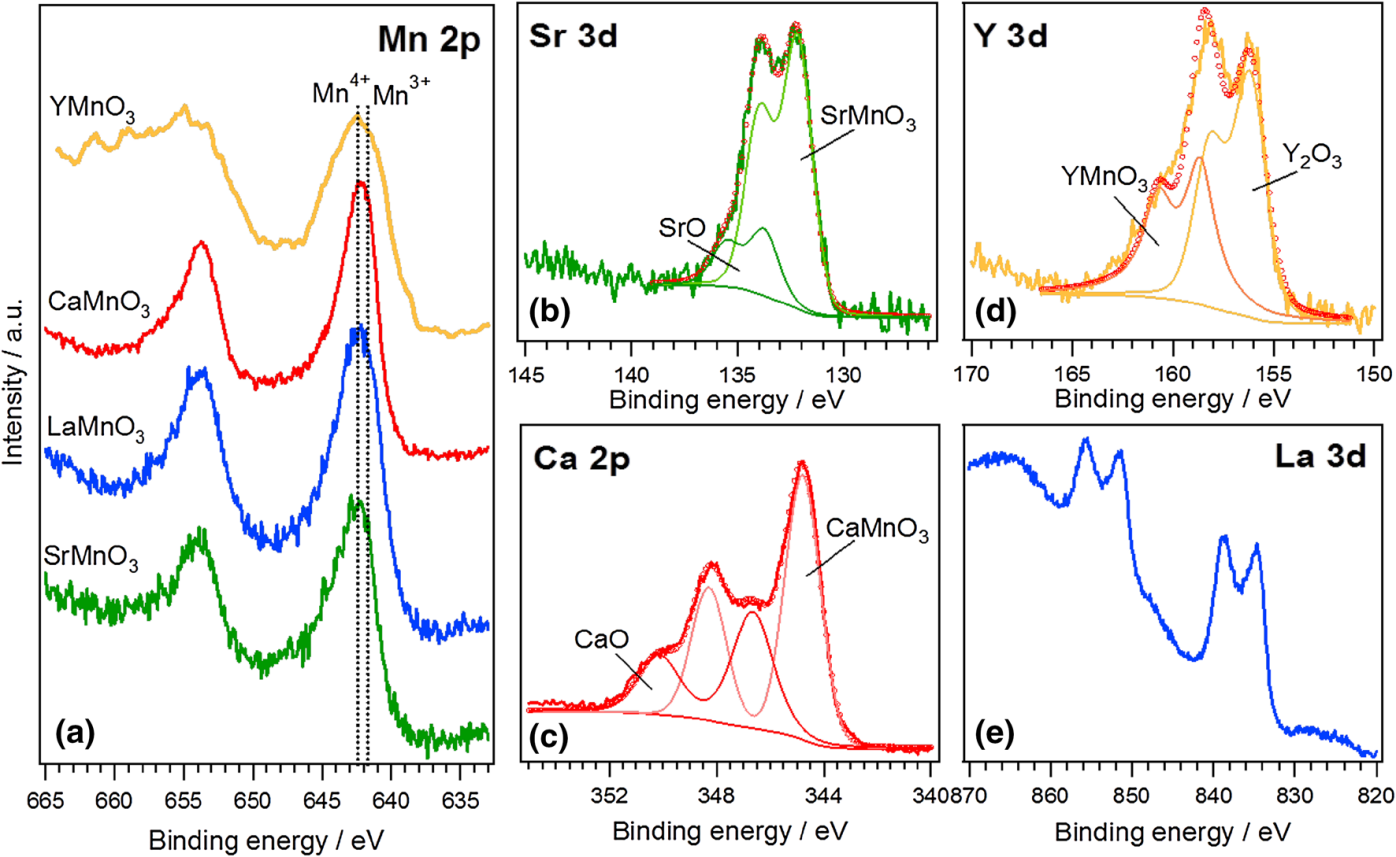
Photoemission spectra of Mn 2*p* (**a**), Sr 3*d* (**b**), Ca 3*d* (**c**), Y 3*d* (**d**) and La 3*d* (**e**) of the various AMnO_3_ oxides taken in normal emission using a non-monochromatic Al Kα X-ray source

Figure 2 shows the XPS spectra for Mn 2*p* and the corresponding A site regions. The Mn 2*p*_5/2_ is observed at around 642.0 eV for all the samples. This broad peak contains contributions from Mn^3+^ (641.9 eV) and Mn^4+^ (642.2 eV) states [30], which are rather difficult to deconvolute. The Sr 3*d* line shows two components; the one at lower BE corresponds to the Sr in the perovskite lattice (132.0 eV), whereas the one at higher BE is attributed to the formation of SrO/Sr(OH)_2_ at the surface (133.8 eV) [31, 32]. Two components are also observed in the Y 3d region, one at 156.2 eV, associated with the formation of Y_2_O_3_, and the another at 158.5 eV related to Y in the lattice of YMnO_3_. La 3d line shows a double splitting due to the interaction between an electron from the oxygen valence band and the empty La 4f level. The La 3d_5/2_ BE is located at 834.4 eV, corresponding to La^3+^ compounds [30, 33]. In the Ca 2p line, the Ca^2+^ species of the perovskite lattice are represented by the component at 345.0 eV, while the Ca 2p_3/2_ component centered at 346.7 eV can be attributed to the formation of CaO in the surface due to the segregation of Ca [14, 34, 35].

The surface ratio of A and Mn sites, as estimated from the Mn 2*p* emission and the regions of the A cations, is summarized in Table S2. A slight A-site surface enrichment is observed in the case of La, Ca and Y, while near stoichiometric ratio is estimated in the case of Sr. In a previous study, we have shown that La has stronger tendency to segregate at the surface than Ca ions [14].

**Fig. 3:**
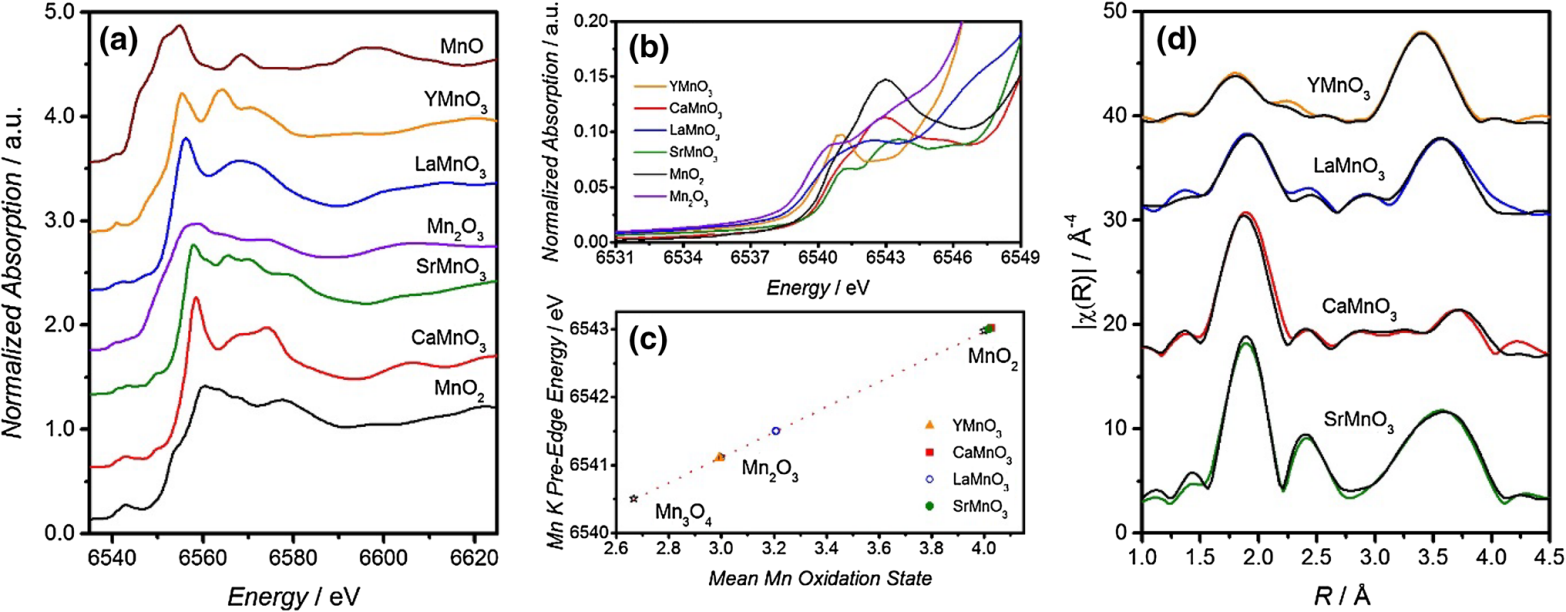
**a** Normalized Mn K-edge XANES spectra of AMnO_3_ (Y, Ca, La, Sr) samples and reference manganese compounds MnO, Mn_2_O_3_ and MnO_2_. **b** Mn K pre-edge features used for quantifying the mean oxidation state. **c** Mean Mn oxidation state as a function of the pre-edge position for the standard MnO, Mn_2_O_3_ and MnO_2_ compounds, as well as for the various AMnO_3_ samples. **d** Data (colour line) and fits (black line) of the FT signal from k^3^-weighted EXAFS signals of YMnO_3_, LaMnO_3_, CaMnO_3_ and SrMnO_3_ until R = 4 Å. Data have been phase-corrected

Figure 3a shows the normalized XANES spectra at the Mn K edge of YMnO_3_, CaMnO_3_, LaMnO_3_ and SrMnO_3_ samples; as well as MnO, Mn_2_O_3_ and MnO_2_ standards. A chemical shift of the main Mn K-edge (considered as the inflection point of the main rise) of 4 eV is observed for Mn_2_O_3_ with respect to MnO_2_, and for LaMnO_3_/YMnO_3_ with respect to CaMnO_3_/SrMnO_3_, as expected for Mn^3+^ and Mn^4+^ [36, 37]. The differences in absolute values between the binary and perovskite-like oxides may be related to geometrical effects, as it is widely acknowledged that the Mn K-edge position can be affected by local distortions of the MnO_6_ octahedra [36, 38–40].

The position of the pre-edge feature as a function of the oxidation state of the Mn is displayed in Fig. 3b. The mean Mn oxidation state was probed from the pre-edge energy position following the approach reported by Croft et al. [41], choosing the minimum point in the derivative spectrum. Figure 3c shows a linear relationship between the pre-edge position and the Mn oxidation state of the standards Mn_3_O_4_, Mn_2_O_3_ and MnO_2_ [41, 42]. The mean Mn oxidation state of the different AMnO_3_ oxides was estimated from this trend, providing values consistent with the composition of the A-site. The manganite oxides containing an alkali metal (2+) at the A-site presented a Mn oxidation state higher (Mn^4+^) than those containing La/Y (Mn^3+^). The slightly higher oxidation state of Mn in LaMnO_3_ could be a reflection of the oxygen vacancies in the structure or the surface segregated manganese sites forming single manganese oxide, and thus, the mean oxidation state would indicate a mixture of Mn^2+^/Mn^3+^/Mn^4+^ sites.

Figure 3d shows the phase-corrected Fourier transform (FT) of the EXAFS region for YMnO_3_, LaMnO_3_, CaMnO_3_ and SrMnO_3_. All of the FT spectra show two strong peaks below 4 Å. The first one at around 2 Å corresponds to the first coordination shell (Mn–O). It is interesting to notice how the amplitude of this peak is smaller in the case of YMnO_3_, for which Mn is only fivefold coordinated with an oxygen atom. The second peak, above 3 Å, is associated with the second shell with Mn–A, Mn–Mn and Mn–O contributions together with multiple scattering paths. The best-fit parameters of the analysis are summarised in Table S3. For a fixed Mn oxidation state, a slight increase of the Mn–O distance is observed as the radius of the A-site cation increases. The fit on *k*-space can be found in Fig. S3.

**Fig. 4:**
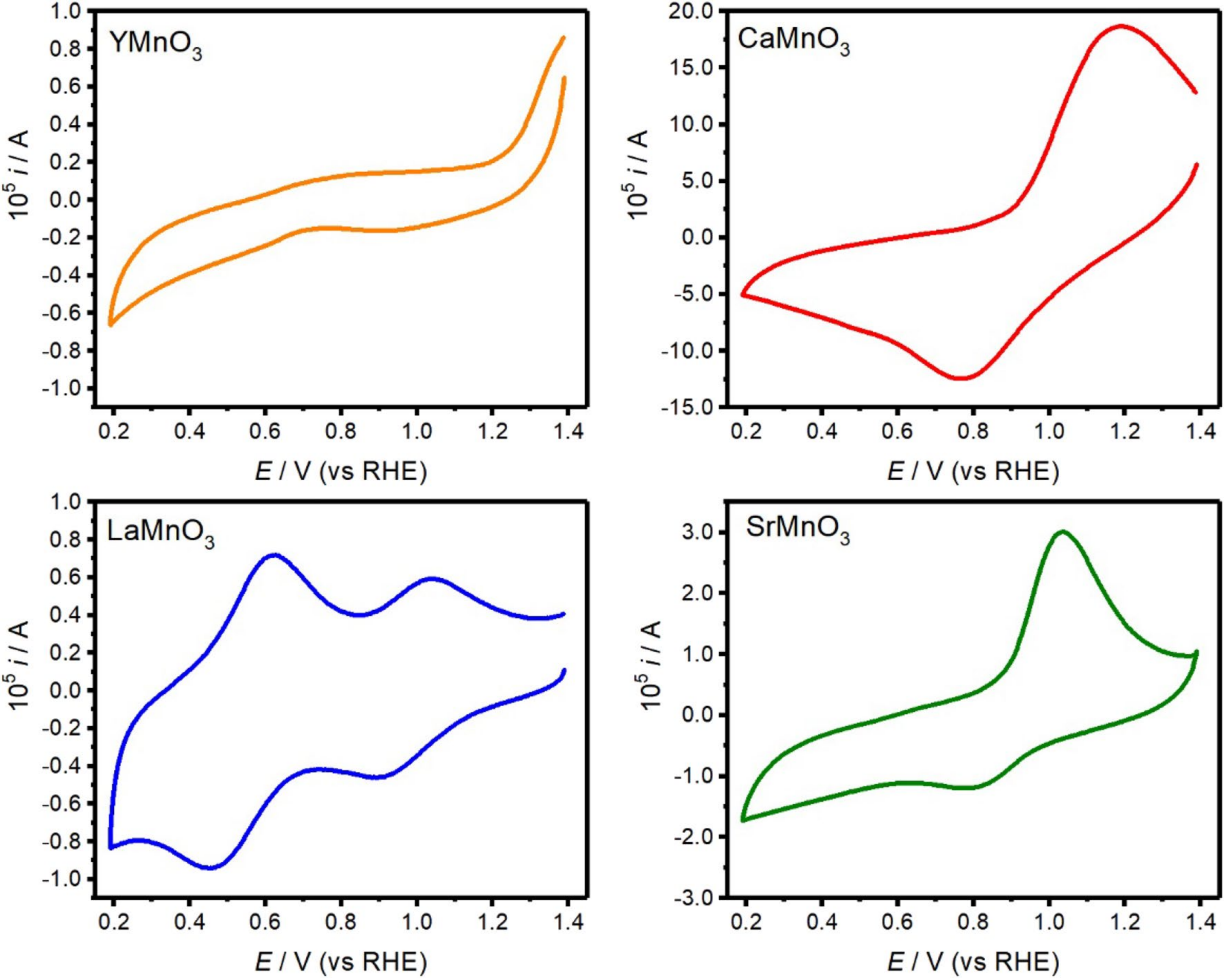
Cyclic voltammograms of YMnO_3_, CaMnO_3_, LaMnO_3_ and SrMnO_3_ supported on mesoporous carbon (Vulcan) in Ar-saturated 0.1 M KOH electrolyte solution at 0.01 V s^−1^. Electrode composition was 250 μg_Oxide_ cm^−2^, 50 μg_Vulcan_ cm^−2^, and 50 μg_Nafion_ cm^−2^

Figure 4 shows cyclic voltammograms (CVs) of the various AMnO_3_ nanostructures with a fix particle loading (250 μg_Oxide_ cm^−2^) in argon-saturated 0.1 M KOH at 0.010 V s^−1^. The CVs are characterised by complex responses associated with the redox properties of surface Mn sites. As the open circuit potential of all of the oxides is located at values more positives than the reduction responses, the initial oxidation state of the Mn site can be taken from the XANES analysis [14]. LaMnO_3_ features two cathodic reduction peaks, located at 0.90 and 0.50 V, related to the reduction of Mn from an oxidation state + 3.2 (as determined in Fig. 3c) to 2+ [14, 15, 18]. The CV of CaMnO_3_ is characterised by a broad reduction peak, ascribed to the reduction of + 4 to + 2 [14, 43, 44]. A similar response is observed from SrMnO_3_, although current magnitude is significantly weaker. Surprisingly, YMnO_3_ shows no clear redox peaks in the voltammetric range investigated. The difference in current responses cannot be simply rationalised in terms of the change in Mn redox state and the mean particle size. For instance, the specific surface area (SSA) of CaMnO_3_ is about three times larger than SrMnO_3_ (see Table S1), while the Mn redox responses is an order of magnitude larger. The rationale for the contrasting behaviour of current responses associated with surface Mn sites remains under investigation.

**Fig. 5:**
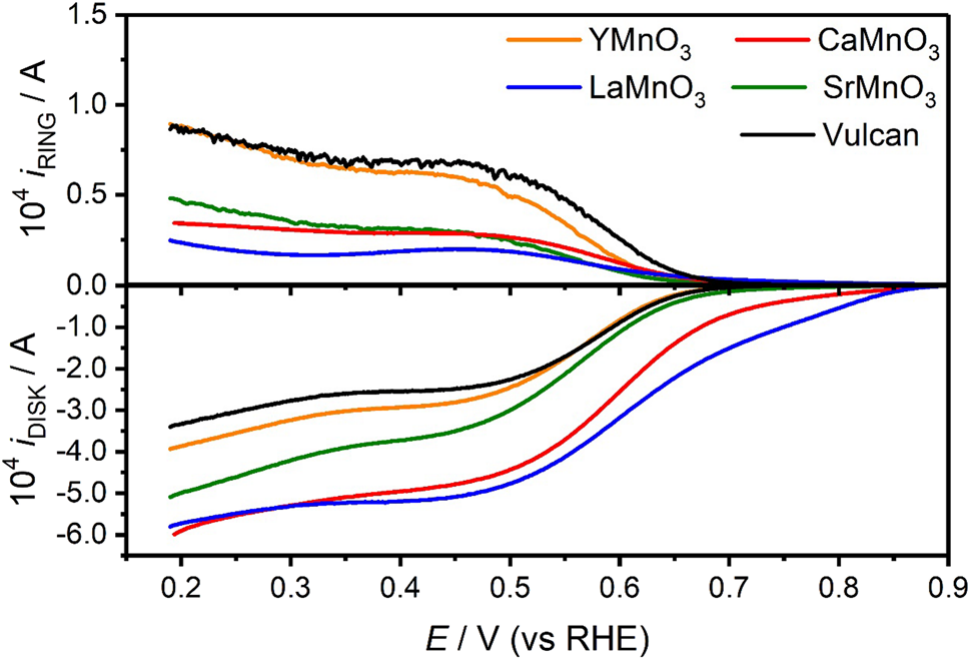
RRDE responses of the various AMnO_3_ (A = Sr, La, Ca, Y) nanoparticles supported at Vulcan layer at 1600 rpm in O_2_-saturated 0.1 M KOH at 0.010 V s^−1^. The Pt ring was held at a constant potential of 1.10 V. The oxide content in each electrode was 250 μg cm^−2^

Figure 5 displays the current measured at the disk (*i*_DISK_), containing a fix carbon-oxide composition, and the Pt ring (*i*_RING_) at 1600 rpm in O_2_-saturated 0.1 M KOH electrolyte. The responses measured for a solely Vulcan carbon electrode have been included as a comparison. It could be clearly seen that LaMnO_3_ and CaMnO_3_ exhibit an ORR onset potential significantly more positive than the Sr and Y manganite. Indeed, the onset potential of SrMnO_3_ and YMnO_3_ is close to the value observed in the absence of oxide, i.e. ORR at the Vulcan support (ca. 0.7 V) [15, 16]. It can also be observed that the diffusional limiting current is smaller for SrMnO_3_ and YMnO_3_, while a larger *i*_RING_ is observed for the latter in the range of 0.2–0.6 V. which is linked to the trend observed in HO_2_^−^ current value. Considering the collection coefficient of the RRDE electrode (*N* = 0.42), the effective number of transferred electrons (*n*) and the hydrogen peroxide yield (% OH_2_^−^) were calculated from the disk and ring currents and displayed in Fig. S5. The trend the OH_2_^−^ yield is YMnO_3_ > SrMnO_3_ > CaMnO_3_ > LaMnO_3_. The effective number of electron ranges from effectively from around 2.6 (YMnO_3_) to 3.6 (LaMnO_3_) in the potential range between 0.2 and 0.6 V. The deviation from the four-electron limit is due to the ORR at the Vulcan support, which effectively goes through a two-electron reaction leading to the production of OH_2_^−^.

The Koutecky–Levich plots shown in Fig. S6 display a complex behaviour associated with the interplay between the two and four electron ORR. The Koutecky–Levich relationship is given by:

5 $$\frac{1}{{{i_{DISK}}}}=\frac{1}{{{i_k}}}+\frac{1}{{{i_L}}}=\frac{1}{{{i_k}}}+\frac{1}{{0.62nAFc{D^{2/3}}{\nu ^{ - 1/6}}{\omega ^{1/2}}}}$$

where *n* is the number of transferred electrons, *A* is the disk geometric area, *F* is the Faraday constant, *c* is the bulk oxygen concentration (1.2 × 10^6^ mol cm^−3^) [45], *D* is the oxygen diffusion coefficient (1.9 × 10^−5^ cm^2^ s^−1^), *ν* is the kinematic viscosity (0.01 cm^2^ s^−1^), and *ω* is the angular rotation of the electrode. *i*_*k*_ and *i*_*L*_ are the kinetically and mass-transport limiting currents, respectively. The slopes observed for LaMnO_3_, CaMnO_3_ and SrMnO_3_ are closer to the limit given by the four-electron reduction process. On the other hand, YMnO_3_ shows a change in slope with the increase of angular rotation rate, reflecting the combination of the two reactions taking place at the oxide and carbon boundaries.

**Fig. 6:**
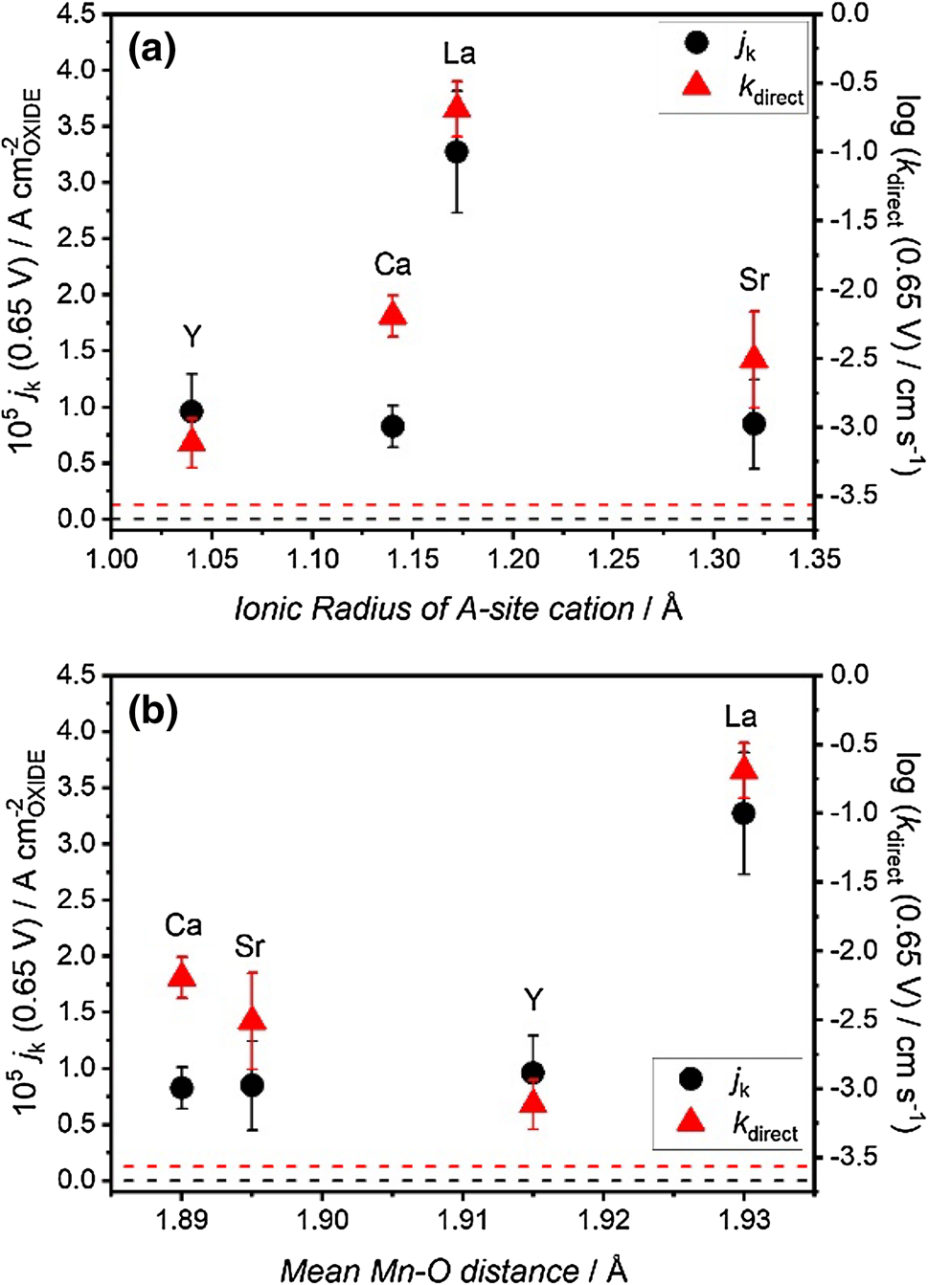
Dependence of the kinetic limiting current density (*j*_k_) and phenomenological electron transfer rate constant for the four-electron step (*k*_direct_) at 0.65 V versus RHE on the ionic radius of the A-site cation (**a**) and the mean Mn–O distance (**b**). The dotted lines correspond to the values of *j*_k_ (black) and *k*_direct_ (red) measured for the carbon support, i.e., in the absence of oxide particles

In order to better illustrate the effect of the A-site on the ORR pathway, we estimated the phenomenological electron transfer rate constant for the four-electron step (*k*_direct_) employing the Damjanovic model [15, 46–49]. Figure 6a, b contrast the value of *k*_direct_ (0.65 V) as a function of the A-site ionic radius and the mean Mn–O bond length obtained from the EXAFS analysis (Table S3). *k*_direct_ effectively represents the rate of conversion to OH^−^, based on the O_2_ flux to the surface (*i*_DISK_) and the rate of conversion to HO_2_^−^ given by *i*_RING_. As this analysis considers mass transport fluxes, the geometric surface area is taken into account as opposed to the effective surface area. It could be observed that as the ionic radii increases, *k*_direct_ goes through a maximum value at A = La (1.93 Å). On the other hand, no clear trend is observed of *k*_direct_ as a function of the mean Mn–O bond distance. The plots also show the values corresponding to the carbon layer (in the absence of catalyst) as dotted line. The values show that Sr and Y based manganite show a modest improvement towards ORR in comparison to Ca and La manganite. Although aspects concerning the effective surface area can play a role in this analysis, the experimental data suggest that A-site ionic radii in the range of 1.15–1.20 Å promote an optimal electronic configuration of the Mn sites for the four-electron ORR.

Figure 6 also show the kinetically limit current density (*j*_k_) at 0.65 V, which is normalised by the specific surface area (SSA) of the oxide. As shown in Table S1, SSA values range significantly from 7.2 ± 1.8 (YMnO_3_) to 51.9 ± 1.8 (CaMnO_3_). Although *j*_k_ exhibits a step increase in the case of LaMnO_3_, this value appears little dependent of the A-site ionic radius as shown in Fig. 6a. On the other hand, a slightly clearer pattern emerges when plotting this data as function of the Mn–O distance (Fig. 6b). A very weak dependence of *j*_k_ is observed with increasing mean Mn–O distance until reaching the value associated with LaMnO_3_. The *j*_k_ baseline value associated with Vulcan is also shown as dotted line, considering the specific surface area of the mesoporous carbon (218 m^2^ g^−1^ [50]). Overall, the analysis show that the overall activity (turnover rate) of the oxide catalysts is larger than the carbon support and significantly increases above a mean Mn–O distance threshold of 1.915 Å.

## Conclusions

Manganite perovskite particles with the general formula AMnO_3_ (A = Y, Ca, La, Sr) were synthesised and assessed as electrocatalysts for the oxygen reduction reaction in alkaline solutions. The synthetic approach led to highly phase pure materials as characterised by XRD and EXFAS, although the different thermochemistry of the oxide phases required various calcination temperatures which led to a range of particle sizes. XPS studies showed that all oxide particles exhibit A-site rich surfaces, although the extent of segregation was weakly dependent of the A-site nature. The kinetics of the ORR as a function of the perovskite composition was quantitatively investigated using rotating ring-disk electrode in O_2_ saturated alkaline solutions. The analysis show that all the perovskite particles catalyse the ORR reaction in comparison to the mesoporous Vulcan support, although the effect of the A-site is rather complex to assess. Our data suggest that A-site with ionic radii in the range of 1.15–1.20 Å strongly promote the four-electron ORR process, while the overall activity increases as the mean Mn–O bond length increases above 1.915 Å. It is also interesting to point out that the Mn sites in the most selective catalysts towards the four-electron process (CaMnO_3_ and LaMnO_3_) are characterised by an octahedral coordination with equal Mn–O distances. Mn is coordinated to 5 oxygen sites with two different Mn–O bond lengths in YMnO_3_, while SrMnO_3_ has Mn sites coordinated to 6 oxygens with two different bond lengths. The electronic configuration of Mn sites in these particles yields different redox behaviour (Fig. 4) and, in agreement with our previous studies, the most active ORR catalysts (LaMnO_3_) show the most positive onset potential for the reduction of surface Mn sites [14, 15].

Although our main conclusions call for further studies correlating structure–activity of these materials, we have uncovered some fascinating trends linking selectivity and ORR with structural parameters such as A-site ionic radius and Mn–O bond length. Theoretical studies linking dioxygen bonding to Mn sites under the various configurations highlighted in these studies can shed more light into the complex activity trends observed of these materials.

## Electronic supplementary material

Below is the link to the electronic supplementary material.

Supplementary material 1 (DOCX 1244 KB)
