# Supplementary material for: AMnO3 (A = Sr, La, Ca, Y) Perovskite Oxides as Oxygen Reduction Electrocatalysts
Source: Top Catal. 2018 Jan 16;61(3):154–61. doi: 10.1007/s11244-018-0886-5 (PMC6413806; doi:10.1007/s11244-018-0886-5)
Supplement: Supplementary file 1 — Supplementary material 1 (DOCX 1244 KB) [file 11244_2018_886_MOESM1_ESM.docx]

**Supporting Information**

AMnO_3_ (A = Sr, La, Ca, Y) perovskite oxides as oxygen reduction electrocatalysts.

V. Celorrio^1, *^, L. Calvillo^2^, G. Granozzi^2^, A.E. Russell^3^, D.J. Fermin^1, *^.

*^1^*School of Chemistry, University of Bristol, Cantocks Close, Bristol BS8 1TS, UK.

*^2^*Dipartimento di Scienze Chimiche, Università di Padova, Via Marzolo 1, 35131 Padova, Italy.

*^3^*School of Chemistry, University of Southampton, Highfield, Southampton, U.K.

Corresponding author:

*Email: [Veronica.Celorrio@bristol.ac.uk](mailto:Veronica.Celorrio@bristol.ac.uk) (V.C.)

*Email: [David.Fermin@bristol.ac.uk](mailto:David.Fermin@bristol.ac.uk) (D.J.F.)


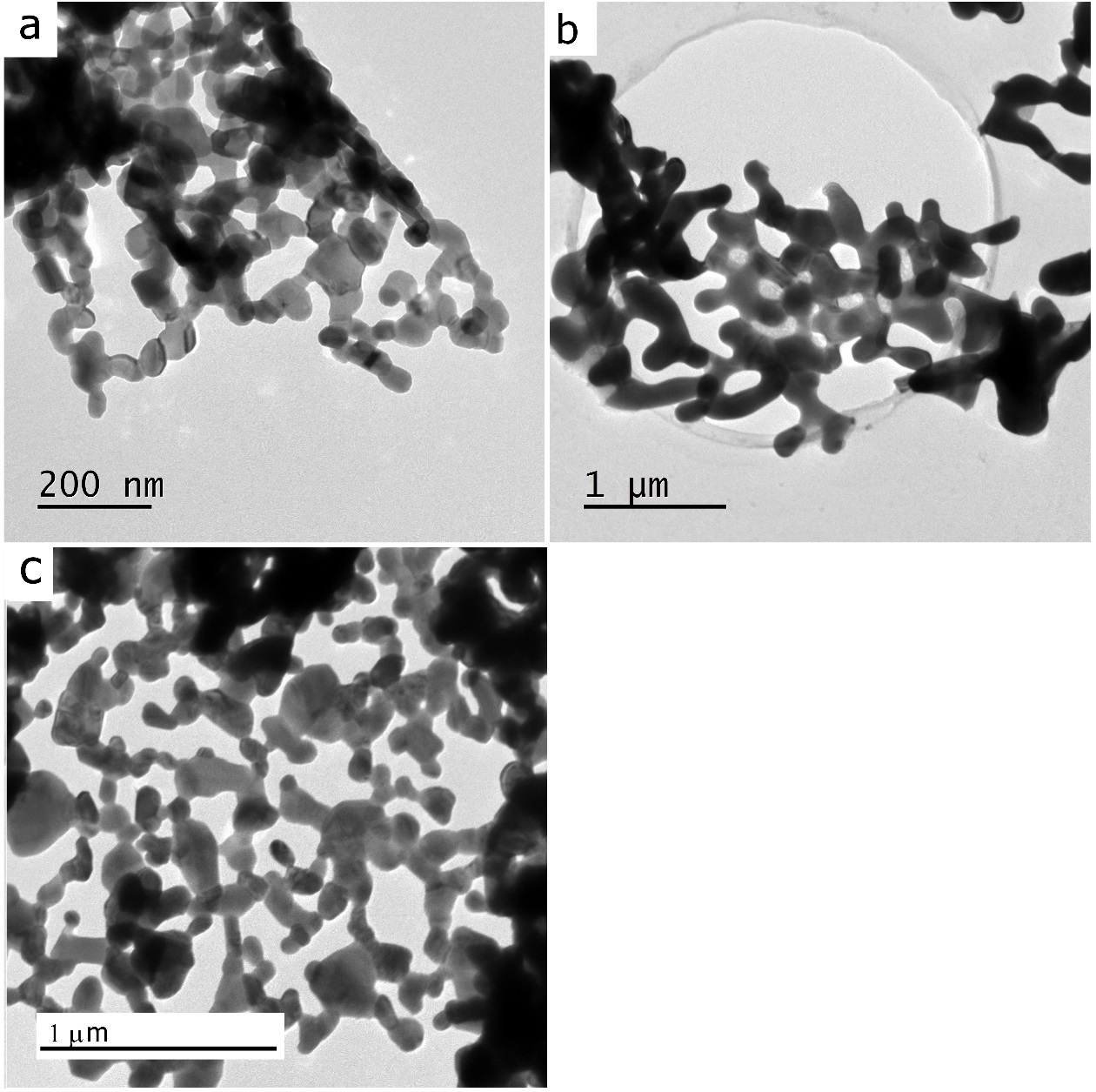


**Fig. S1** Characteristic TEM images LaMnO_3_ (a) and YMnO_3_ (b) and SrMnO_3_ (c) oxide particles.


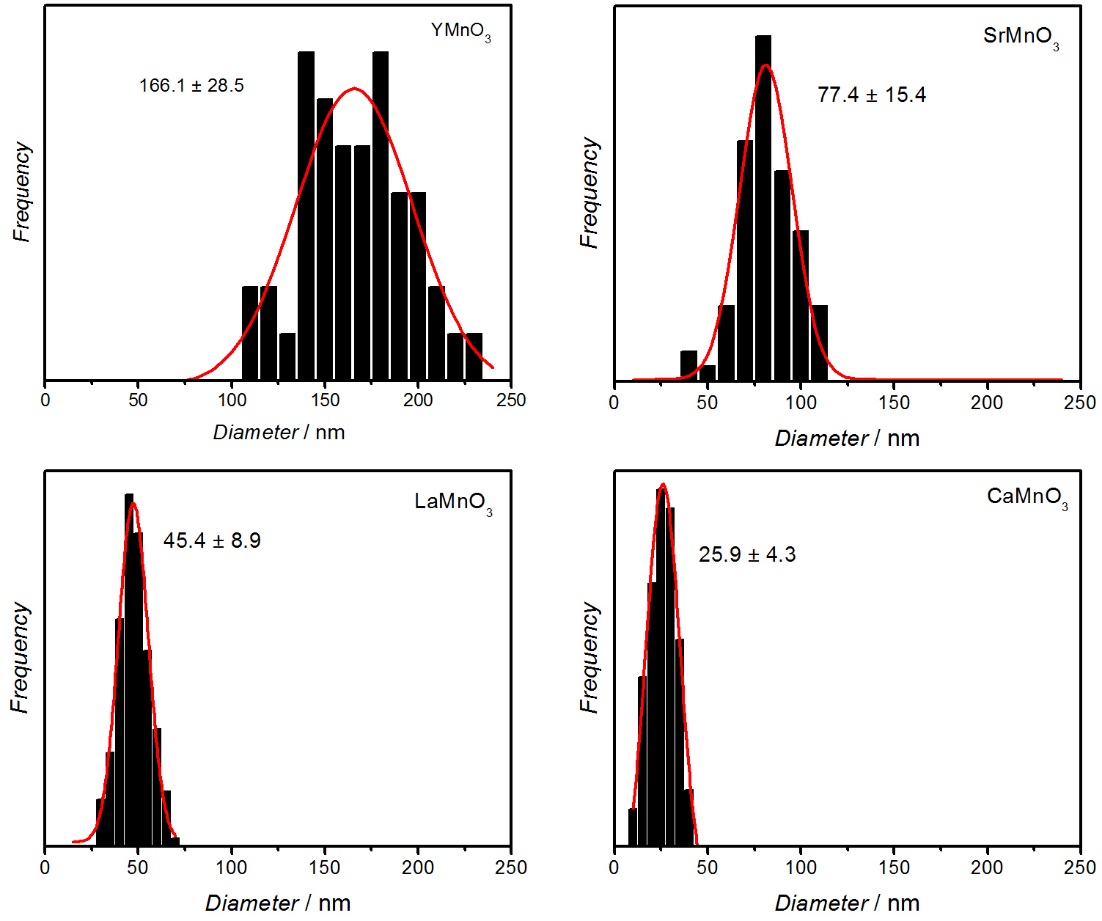


**Fig. S2** Particle size distributions for SrMnO_3_, LaMnO_3_, CaMnO_3_ and YMnO_3_ oxides.


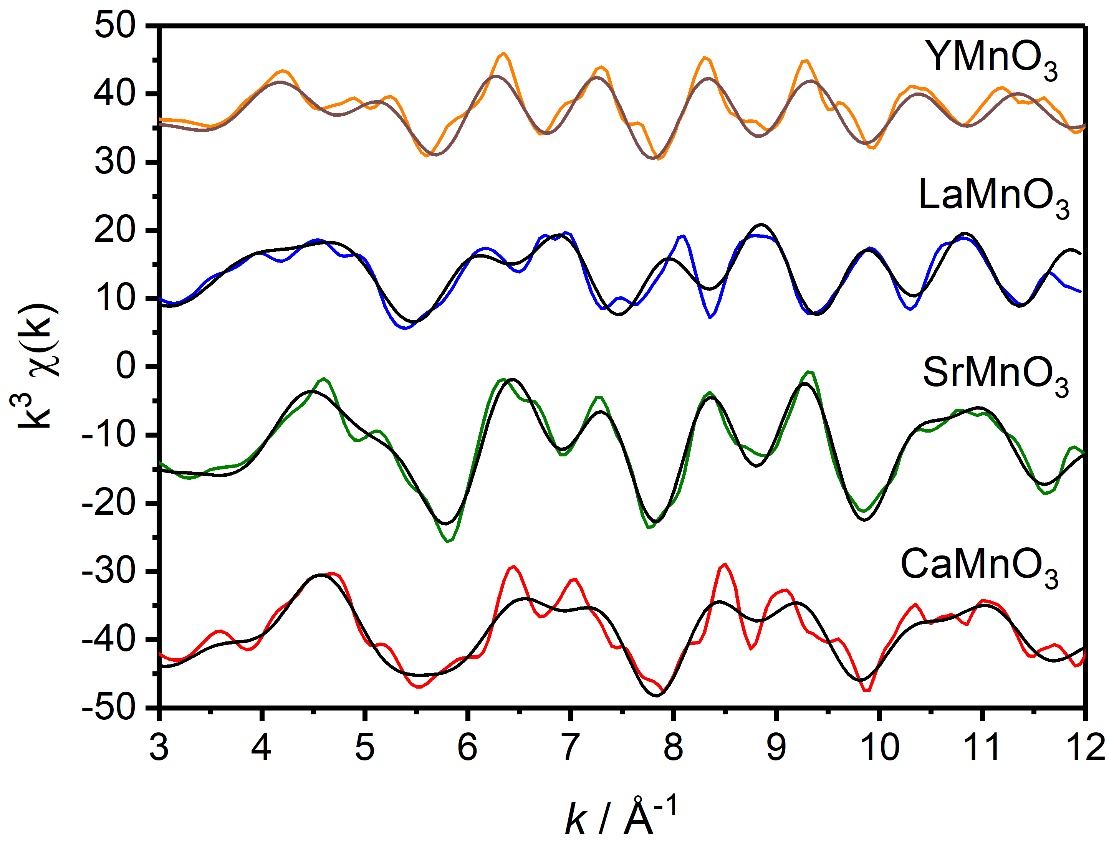


**Fig. S3** Data (colour line) and fits (black line) of the k^3^-weighted EXAFS signals in *k*-space for YMnO_3_, LaMnO_3_, CaMnO_3_ and SrMnO_3_. Data was fitted between 3-12 Å^-1^.


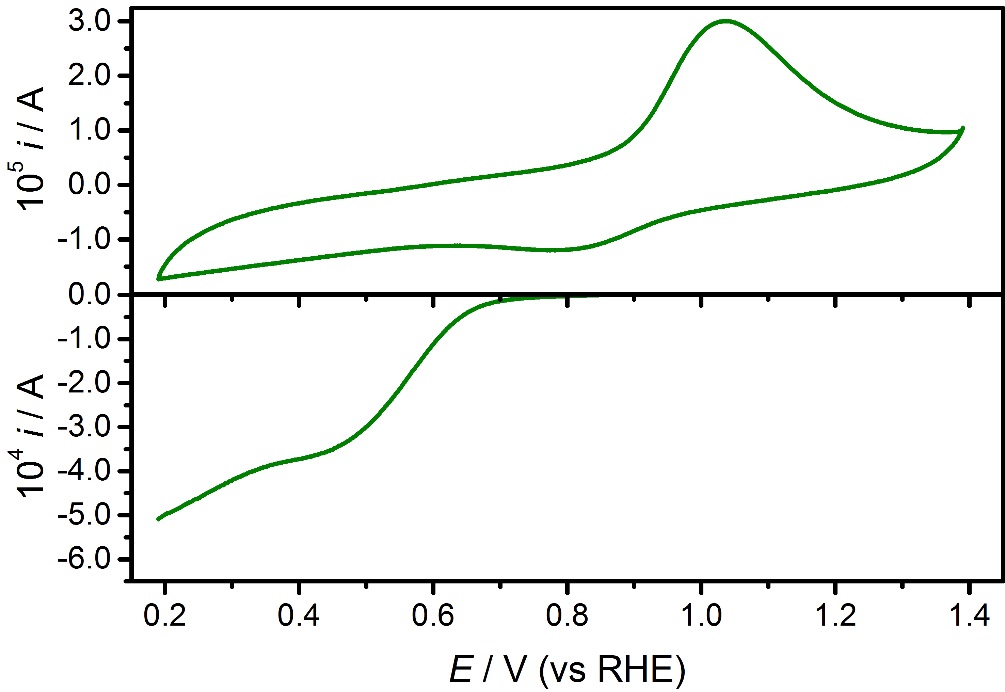


**Fig. S4** Overlay of the cyclic voltammograms measured in Ar-saturated 0.1 M KOH (top
panels) and the disc current recorded at 1600 rpm in O2-saturated 0.1 M KOH for nanoparticles.


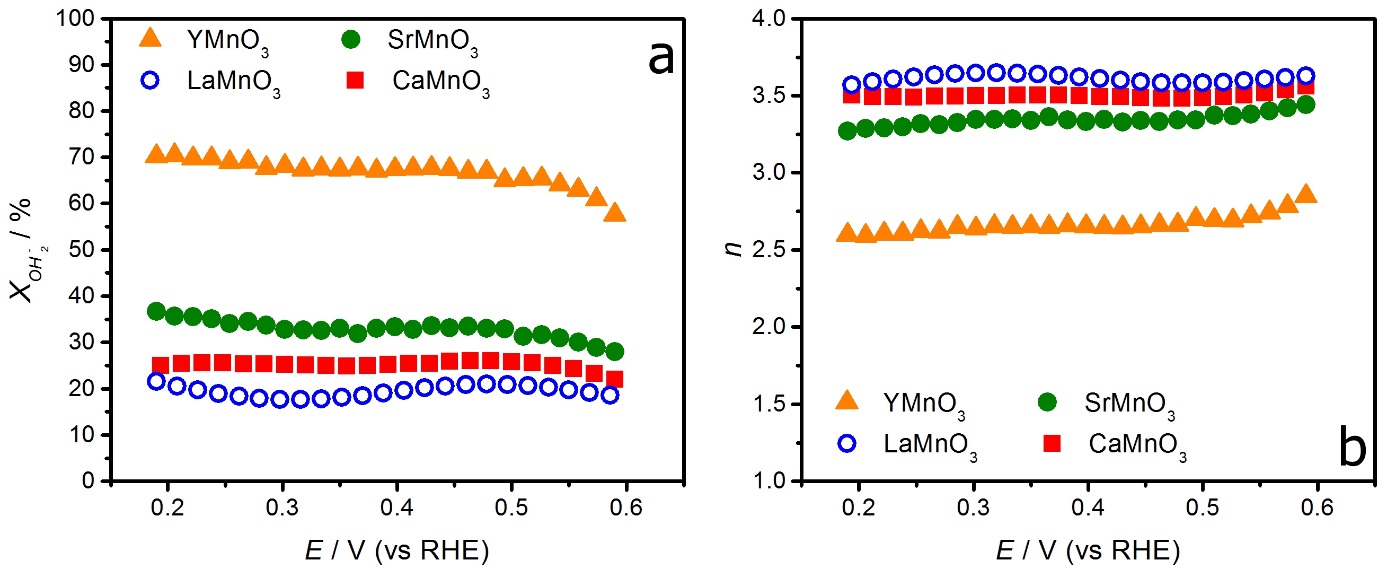


**Fig. S5** $OH_{2}^{-}$ yield (a) and number of electrons transferred, *n* (b) calculated from
measurements of *i*_RING_ and *i*_DISK_ for SrMnO_3_, LaMnO_3_, CaMnO_3_ and YMnO_3_ electrodes in O_2_-saturated 0.1 M KOH at 1600 rpm. The potential range correspond to the diffusion limiting region.

$X_{{HO}_{2}^{-}}=\frac{{2i_{RING}}/N}{i_{DISK}+{i_{RING}}/N}\times100$ Equation S1

$n=\frac{4i_{DISK}}{i_{DISK}+{i_{RING}}/N}$ Equation S2

where *N* is the collection coefficient (N = 0.42).


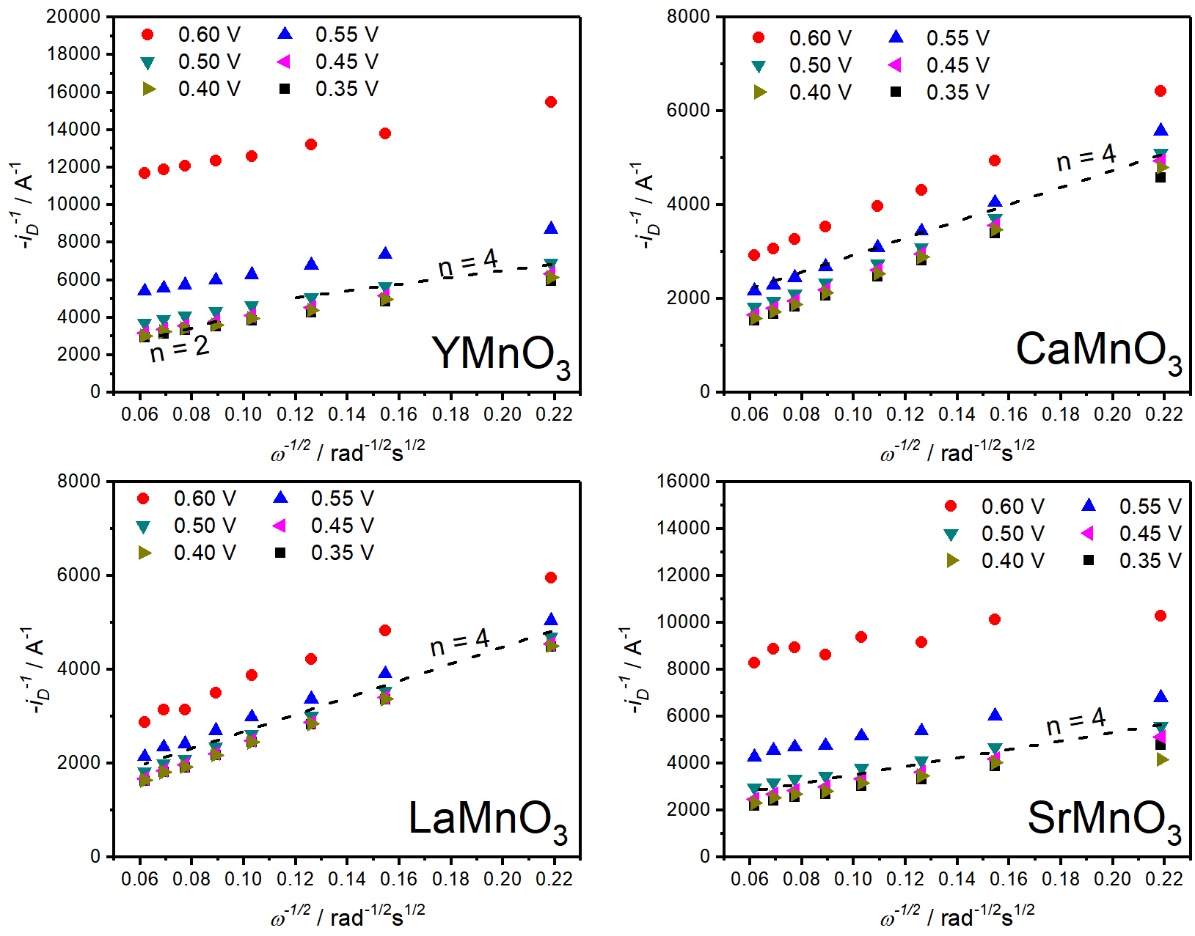


**Fig. S6** Koutecky-Levich plots for ORR at Vulcan supported YMnO_3_ (a), CaMnO_3_ (b), LaMnO_3_ (c) and SrMnO_3_ (d) nanoparticles in O_2_-saturated 0.1 M KOH solution at different potentials. Dashed line shows the theoretical slopes corresponding to the 2e^-^ and 4e^-^ ORR process (note this is not a fit to the data).

**Table S1.** Specific surface area (SSA) calculated assuming that the oxide nanoparticles are spherical,

SSA (m^2^ g^-1^) = 6×10^3^/(*ρ*×*d*)

where *d* is the mean particle size determined by TEM (nm), and *ρ* is the theoretical density (g cm^-3^).

|  | *ρ* / g cm^-3^ | *d /* nm | *SSA* / m^2^ g^-1^ |
| --- | --- | --- | --- |
| YMnO_3_ | 5.14 | 166.1 ± 28.5 | 7.2 ± 1.8 |
| CaMnO_3_ | 4.59 | 25.9 ± 4.3 | 51.9 ± 12.2 |
| LaMnO_3_ | 6.57 | 45.4 ± 8.9 | 20.9 ± 5.8 |
| SrMnO_3_ | 5.40 | 77.4 ± 15.4 | 14.9 ± 4.2 |

**Table S2.** A:Mn atomic ratio on the surface of the LaMnO_3_ samples calculated from XPS. The surface composition of the samples was obtained from the Mn 2p, Ca 2p, Sr 3d, Y 3d, and La 3d peak regions taking into account the corresponding sensitivity factors. The estimated error is ±2.

|  | A : Mn |
| --- | --- |
| YMnO_3_ | 56 : 44 |
| CaMnO_3_ | 61 : 39 |
| LaMnO_3_ | 64 : 36 |
| SrMnO_3_ | 49 : 51 |

**Table S3.** Relative energy shift and the best fit results from the structural analysis of the different AMnO_3_ (A = Y, Ca, La, Sr) at the Mn K-edge. N is the coordination number, R_Mn-O_ is the interatomic distance and σ^2^ is the Debye-Waller factor. R_f_ is the R-factor, which represents the relative error of the fit and data.

|  | Shell | N | R / Å | σ^2^ x 10^3^ / Å^2^ | ΔE_0_ / eV | R_f_ |
| --- | --- | --- | --- | --- | --- | --- |
| YMnO_3_ | Mn-O_1_ | 2 | 1.85 ± 0.03 | 2.9 ± 2.5 | -7.0 ± 0.1 | 0.008 |
|  | Mn-O_2_ | 3 | 1.98 ± 0.03 | 8.4 ± 5.4 |  |  |
|  | Mn-Y | 6 | 3.33 ± 0.03 | 9.8 ± 2.0 |  |  |
|  | Mn-Mn_2_ | 6 | 3.51 ± 0.03 | 6.5 ± 2.0 |  |  |
| CaMnO_3_ | Mn-O_1_ | 6 | 1.89 ± 0.01 | 2.8 ± 1.0 | -4.8 ± 1.3 | 0.019 |
|  | Mn-Ca | 8 | 3.21 ± 0.03 | 14.8 ± 2.2 |  |  |
|  | Mn-Mn_2_ | 6 | 3.73 ± 0.03 | 8.9 ± 2.1 |  |  |
|  | Mn-O_1_ | 3 | 1.88 ± 0.03 | 2.1 ± 1.2 | -1.6 ± 1.4 | 0.017 |
|  | Mn-O_2_ | 3 | 1.91 ± 0.03 | 2.1 ± 1.2 |  |  |
| SrMnO_3_ | Mn-Mn_1_ | 1 | 2.50 ± 0.03 | 2.2 ± 1.9 |  |  |
|  | Mn-Sr | 4 | 3.33 ± 0.02 | 5.0 ± 1.6 |  |  |
|  | Mn-Mn_2_ | 3 | 3.75 ± 0.01 | 4.9 ± 1.7 |  |  |
|  | Mn-O_1_ | 6 | 1.93 ± 0.02 | 6.8 ± 1.8 | -5.3 ± 2.3 | 0.016 |
| LaMnO_3_ | Mn-La_1a_ | 2 | 3.23 ± 0.06 | 4.8 ± 1.0 |  |  |
|  | Mn-La_1b_ | 6 | 3.36 ± 0.02 | 4.8 ± 1.0 |  |  |
|  | Mn-Mn_2_ | 6 | 3.81 ± 0.06 | 21.3 ± 5.7 |  |  |

**Table S4.** Phenomenological electron transfer rate constant for the four-electron step (*k*_direct_) and kinetically limiting current normalised by and the area of oxide present at the electrode (*j*_k_) at 0.65 V vs RHE.

|  | *Ionic Radius of A-site Cation* / Å | *Mean Mn-O distance* / Å | *j*_k_ / A cm^-2^_OXIDE_ | *k*_direct_ / cm s^-1^ |
| --- | --- | --- | --- | --- |
| YMnO_3_ | 1.04 | 1.915 | (9.6 ± 3.2) · 10^-6^ | (7.7 ± 2.1) · 10^-4^ |
| CaMnO_3_ | 1.14 | 1.890 | (8.3 ± 1.8) · 10^-6^ | (6.5 ± 1.0) · 10^-3^ |
| LaMnO_3_ | 1.17 | 1.930 | (32.7 ± 5.4) · 10^-6^ | (2.0 ± 0.7) · 10^-1^ |
| SrMnO_3_ | 1.32 | 1.895 | (8.5 ± 3.9) · 10^-6^ | (3.1 ± 0.8) · 10^-3^ |
